# Occupational Hearing Loss in the Intensive Care Unit: A Comparative Study Between ICU and Non‐ICU Nursing Staff

**DOI:** 10.1111/nicc.70697

**Published:** 2026-09-24

**Authors:** Ziwei Song, Pyoung Jik Lee, Li Bin, Thomas Hampton

**Affiliations:** ^1^ Acoustics Research Unit School of Architecture, University of Liverpool Liverpool UK; ^2^ School of Design and Humanities Chongqing University of Science & Technology Chongqing China; ^3^ Nanchuan District People's Hospital Chongqing China; ^4^ Department of Eye and Vision Sciences, Institute of Life Course and Medical Sciences University of Liverpool Liverpool UK; ^5^ NHS University Hospitals of Liverpool Liverpool UK

**Keywords:** digits‐in‐noise, intensive care unit, noise, occupational hearing loss, pure‐tone audiometry

## Abstract

**Background:**

Occupational exposure to sustained high‐noise levels in intensive care units (ICUs) could pose significant auditory health risks for healthcare professionals.

**Aim:**

This investigation evaluates the prevalence and characteristics of hearing impairment among nursing staff working in an intensive care unit (ICU) versus non‐ICU environments within Chinese healthcare facilities.

**Study Design:**

A cross‐sectional study was conducted at a hospital in Chongqing, China. Participants underwent comprehensive audiological assessments including pure‐tone audiometry (PTA) and Chinese digits‐in‐noise (DIN) (testing for speech reception thresholds (SRT)). Statistical analyses employed univariate and multivariate logistic regression models to identify independent predictors of hearing impairment.

**Results:**

A total of 93 registered nurses (45 ICU and 48 non‐ICU) were included in the final analysis. ICU nurses demonstrated significantly worse hearing thresholds compared to non‐ICU counterparts, particularly at high frequencies (4–8 kHz). Approximately 20% of ICU nurses exhibited hearing loss (> 25 dB HL) at high frequencies, compared to 10% in non‐ICU settings. Working in ICU environments (adjusted OR = 2.82, 95% CI: 1.25–6.35), working 50–60 h per week (adjusted OR = 4.15, 95% CI: 1.18–14.60), and working more than 60 h per week (adjusted OR = 5.85, 95% CI: 1.55–22.10) emerged as significant independent predictors of hearing impairment. Speech reception thresholds were significantly poorer among ICU nurses (*p* < 0.05), with PTA thresholds showing a strong correlation with SRT performance (*r* = 0.824, *p* < 0.001). Multiple regression analysis identified 2 kHz as the most critical frequency contributing to speech understanding in noise for both groups.

**Conclusions:**

This study provides compelling evidence of elevated hearing impairment risk among ICU nurses compared to non‐ICU nursing staff in China. These findings highlight the urgent need for targeted occupational health interventions and hearing conservation programmes for nursing staff working in high‐noise environments.

**Relevance to Clinical Practice:**

This study emphasises the necessity of targeted occupational health measures for ICU nurses, particularly those with prolonged working hours. Clinical protocols should prioritise monitoring high‐frequency hearing thresholds and incorporating speech‐in‐noise assessments to detect functional deficits. Furthermore, implementing workload management strategies is essential to mitigate the dose–response risk of noise exposure and preserve nurses' long‐term auditory health.

## Impact Statements

- What is known about the topic
  ○ Occupational noise exposure is a well‐documented cause of hearing impairment in industrial sectors like manufacturing and construction.
  ○ Intensive Care Units (ICUs) are known to be high‐noise environments, frequently exceeding the World Health Organization's recommended levels.
- What this paper adds
  ○ This study provides objective evidence that ICU nurses in China have significantly worse hearing thresholds at high frequencies (4–8 kHz) compared to their non‐ICU counterparts.
  ○ Approximately 20% of ICU nurses exhibited high‐frequency hearing loss, representing a risk level nearly double that of nurses in other departments.
  ○ Working more than 50 h per week was identified as a critical independent risk factor, suggesting a dose–response pattern between extended shifts in noisy environments and auditory damage.

## 1 Introduction and Background

Occupational noise exposure constitutes a significant yet often overlooked public health issue. Although the occupational noise association with hearing impairment is well‐documented in industrial settings such as manufacturing [1], and construction [2], and across various occupational sectors [3], evidence from non‐traditional high‐noise environments remains scarce. Healthcare workers, particularly nurses, represent a susceptible yet under‐researched population in this context. In addition, prior occupational noise exposure studies have demonstrated a higher prevalence of tinnitus among those with noise‐induced hearing loss, and this symptom could independently or synergistically impact on workplace performance [4].

Intensive care unit (ICU) nurses experience chronic occupational exposure to elevated noise levels, often working extended shifts where sound pressure levels exceed recommendations. For instance, Che et al. [5] established that only 35% of nurses in China work < 40 h per week, and research from other fields has suggested long work hours may compound noise‐induced hearing risks [6, 7]. Previously, Messano, Petti [8] documented a 30% incidence of hearing problems in dentists, especially those with 10 or more years of practice who routinely use potentially noisy equipment. Furthermore, given that the nursing workforce is predominantly female in China [9], additional data on this issue could have important equity implications, as emerging evidence suggests gender may modify susceptibility to both age‐related and noise‐induced hearing loss [10].

The characterisation of hearing impairment in this context requires a multifaceted approach. Alongside standard pure‐tone audiometry (PTA), functional assessments like the digits‐in‐noise (DIN) test are vital for detecting early deficits [11]. This is particularly true in China, where a rapidly developing healthcare system faces unique operational pressures. Despite high‐noise levels in ICUs, no studies have directly compared objective hearing outcomes between ICU and non‐ICU nurses. In particular, knowledge gaps persist regarding: (1) the comparative prevalence of hearing impairment between ICU and non‐ICU nurses; (2) the association of occupational factors (department and work hours) with objective hearing measures; and (3) the correlation between pure‐tone thresholds and speech‐in‐noise performance.

## 2 Aims

This study aims to evaluate the prevalence and characteristics of hearing impairment among nursing staff working in ICU versus non‐ICU environments within Chinese healthcare facilities. By comparing PTA and DIN test results, the study seeks to quantify and compare occupational hearing loss between these two clinical settings. The underlying hypothesis suggests that ICU nurses exhibit worse hearing thresholds and poorer speech reception thresholds in noise relative to their non‐ICU counterparts.

## 3 Design and Methods

### 3.1 Setting and Sample

This study was conducted at a district‐level tertiary general hospital in China from January 2023 to March 2023. Participants were recruited via internal announcements (e.g., posted the advertisement) and departmental meetings. The advertisement, along with the participant information sheet, was also circulated to all nurses via the hospital's administration team. Nurses who were interested in participating contacted the research team prior to scheduling of the hearing assessments. The non‐ICU group consisted of nurses recruited from general medical wards, including gastroenterology, respiratory medicine and geriatric wards, who were matched to the ICU group based on years of clinical experience. This sample size exceeds the 45 participants required to achieve a statistical power of 0.8 for an independent‐sample *t*‐test, as calculated using G*Power software. All participants were screened for the presence of any previous or existing ear and hearing problem with the following inclusion criteria: (a) no previous history of any ear infection; (b) no history of any trauma to the head and neck region; (c) no systemic illness or history of any ototoxic drug exposure; and (d) no history of any nerve injury or trauma involving the central or peripheral nervous system. In addition, otoscopic examination was conducted to exclude participants with any active or existing ear diseases.

### 3.2 Data Collection Tools and Methods

PTA was conducted using a calibrated audiometer (GSI AudioStar Pro) in a soundproof room (ambient noise < 30 dBA). Threshold was estimated using a modified Hughson–Westlake (five‐up‐ten‐down) method for the air and bone conduction. Participants wore headphones (TDH‐39) over ear and responded to pure tones generated from an adjacent observation room. Changes in overall hearing sensitivity were assessed based on response alterations for each frequency.

The speech testing was performed using the Chinese version of the DIN test via the ‘HearWHO’ app (2021). Spoken digit triplets were presented against broad band masking noise (long‐term average speech spectrum noise, LTASS). Participants were instructed to use headphones (Sony MDRZX110AP ZX) and select a comfortable volume, whereas digit triplets were presented without masking noise. After a five‐triplet familiarisation period, the SRT for each ear was determined using a set of 17 triplets. The initial signal‐to‐noise ratio (SNR) was set at 0 dB, and participants were asked to repeat the digits they heard. Correctly or incorrect answers led to a decrease or increase in the SNR by 2 dB [12]. SRT results were categorised as ‘Good’, ‘OK’ or ‘Needs Help’, based on specific cut‐off values. Similar to the English language set, the cut‐offs for ‘Good’ were SRTs ≤ −15.2 dB SNR, between −15.1 and −12.5 dB SNR for ‘OK’ hearing and > −12.5 dB SNR for ‘Needs Help’. These cut‐offs were based on the normative dataset presented in the previous study [13].

To control for potential confounding variables affecting hearing thresholds, detailed data on participants' demographic and health characteristics were collected, including age, gender, working hours and working years. These variables were subsequently entered as covariates in the multivariate regression analysis to adjust for their potential confounding effects on the association between occupational noise exposure and hearing threshold. Additionally, the history of ototoxic medication use was recorded, and participants with active ear diseases or conditions known to independently cause hearing loss were excluded prior to analysis to minimise physiological confounding.

### 3.3 Data Analysis

Statistical analyses were mainly performed using IBM SPSS Statistics 25 (IBM Corp., Armonk, NY, USA). The normality of data distribution was assessed using the Shapiro–Wilk test. Spearman's rank correlation coefficient was employed to examine the relationships between PTA and SRT results. To evaluate the impact of hearing loss on speech comprehension, the frequency‐weighted average pure‐tone hearing thresholds were calculated using the formula: (HT500 + 2·HT1000 + 2·HT2000 + HT4000)/6 [14]. Additionally, PTA thresholds at 8 kHz were computed to assess high‐frequency hearing loss. Given the limited number of positive events, univariate and multivariate associations between elevated hearing thresholds (> 25 dB HL) and nurses' socio‐demographic and occupational characteristics (age, working hours, working years) were examined using Firth's penalised‐likelihood logistic regression, which reduces small‐sample bias [15]. These analyses were conducted using the logistf package (version 1.26.1) in R (version 4.5.2). Results were presented as odds ratios (OR) with 95% confidence intervals (CI). An independent‐samples *t*‐test was utilised to compare hearing thresholds between ICU and non‐ICU nurses. Statistical significance was defined as *p* < 0.05 for all analyses.

### 3.4 Ethical Considerations

The study protocol was approved by the Central Ethics Committee (No. 11995) of the University of Liverpool on December 13, 2022. Written informed consent was obtained from all participants, and all data were anonymised.

## 4 Results

A total of 93 nurses (45 from the ICU and 48 from non‐ICU environments) were included in the final analysis. Table 1 presents the socio‐demographic and professional characteristics of the participants. Among participants, 89% were female and 11% were male, which is typical of the general healthcare workforce in China. For instance, Li et al. [16] reported that the proportion of female health professionals in China was 72.4% in 2020. There were similar years of experience for ICU (mean 7.2, SD 3.1) and non‐ICU nurses (mean 6.3, SD 3.6). Participants' ages ranged from 20 to 58 years (mean 33.1, SD 6.8), with the majority having worked in hospitals for more than 5 years (58.1%).

**TABLE 1:** Socio‐demographic and professional characteristics of the participants.

Personal characteristics	ICU (*N* = 45)	Non‐ICU (*N* = 48)	Total (*N* = 93)
Gender			
Male	2	8	10
Female	43	40	83
Age (years old)			
20–30	15	21	36
31–40	27	20	47
> 40	3	7	10
Years of working			
< 1	0	2	2
1–2	0	9	9
3–5	8	13	21
6–10	31	23	54
> 10	6	1	7
Hours of working per week			
< 40	0	0	0
40–49	30	33	63
51–59	7	11	18
≥ 60	8	4	12

Abbreviation: ICU, intensive care unit.

The average PTA hearing thresholds of each frequency in both ears are listed in Table S1. A paired sample *t*‐test revealed that the binaural hearing was symmetrical, and the difference in hearing thresholds between the left and right ears was not statistically significant (*t* = 0.03–0.63 and *p* = 0.53–0.98) across all frequencies for both groups (see Table S1). Therefore, the average hearing threshold of both ears at each frequency was used for subsequent analyses.

**FIGURE 1:**
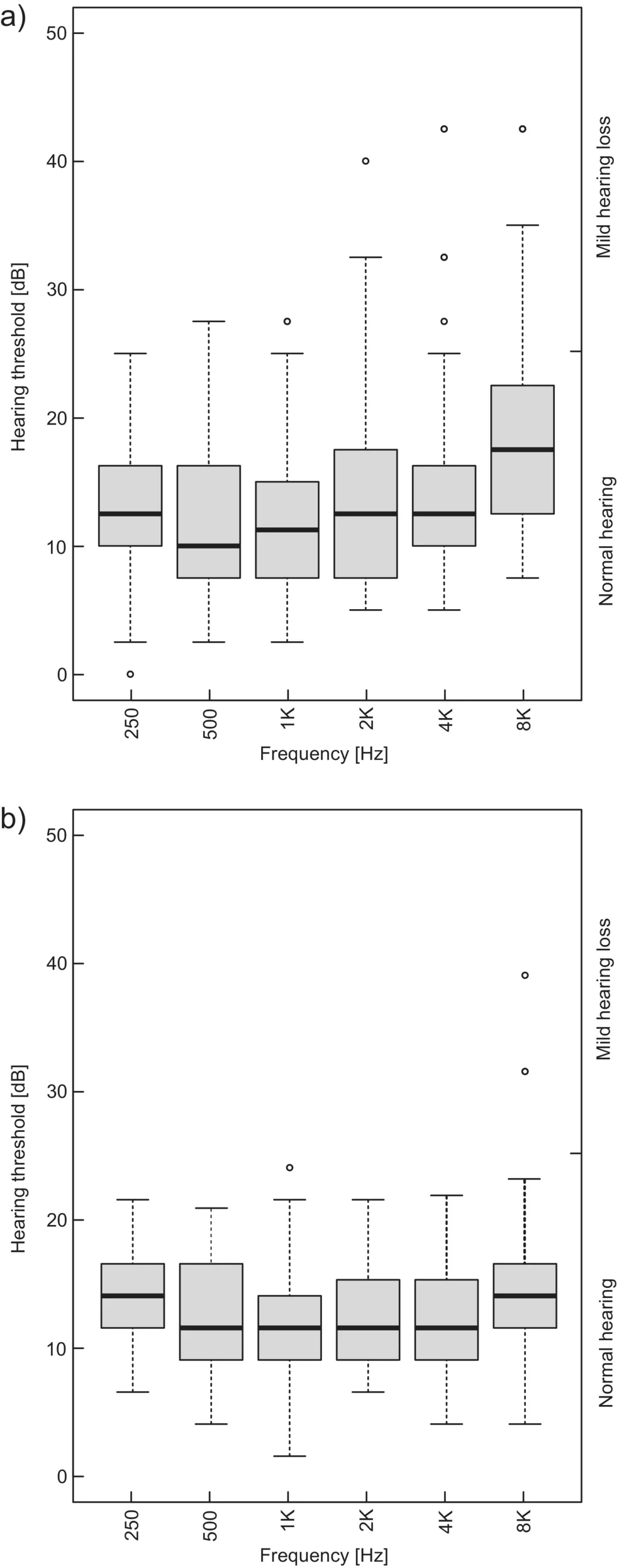
Hearing thresholds among two nurse groups at different frequencies: (a) ICU nurses and (b) non‐ICU nurses. The upper and lower boundaries of the boxes indicate the 75th and 25th percentiles, the bars in the middle of the boxes indicate the medians. The whiskers show the minimum and maximum (dB: Decibel and Hz: Hertz).

The hearing thresholds between two groups at different frequencies are plotted in Figure 1. Both ICU nurses and non‐ICU nurses had normal hearing thresholds (< 25 dB HL) at frequencies below 1 kHz. In general, ICU nurses reported increased hearing thresholds with increased frequencies, and the difference between hearing threshold at frequencies above 4 kHz and hearing threshold at frequencies below 1 kHz was statistically significant (*p* < 0.01). Remarkably, approximately 20.0% of ICU nurses and 10.4% of non‐ICU nurses experienced hearing loss (> 25 dB HL) at high frequencies (4 kHz and 8 kHz).

**FIGURE 2:**
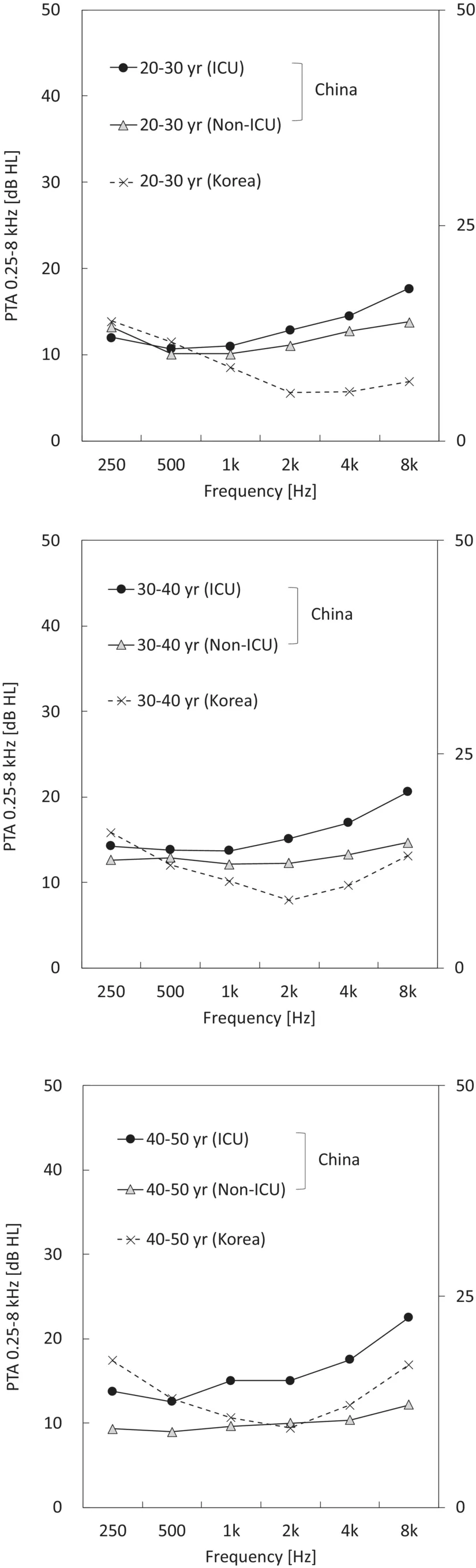
Comparisons of hearing thresholds between the current study and the Korean study for different age bands (top: 20–30 years, middle: 31–40 years and bottom: > 40 years) (PTA: Pure‐tone audiometry, HL: Hearing loss, dB: Decibel and Hz: Hertz).

The hearing thresholds of nurses measured in this study were compared with those reported in previous studies. For these, the PTA results were categorised by age groups (20–30 years, 31–40 years and > 40 years). First, the hearing thresholds were compared with the International Organization for Standardization standard [17], which provides statistical distribution of hearing thresholds based on age and gender. Both ICU and non‐ICU nurses showed poorer hearing thresholds compared to the ISO 7029 estimates for their respective age groups (see Figure S1). However, the ISO 7029 was established based on data collected from European and North American populations in the 1950s–1970s, which may be outdated and not representative of other ethnic groups. Secondly, the PTA‐based hearing thresholds across different age groups were compared with data from Korean adults [18]. As shown in Figure 2, ICU nurses reported similar hearing thresholds at low frequencies but exhibited worse hearing thresholds at high frequencies compared to those reported in the Korean study.

Univariate and multivariate logistic regression analyses were conducted to examine the impact of socio‐demographic and working characteristics on hearing thresholds for both ICU and non‐ICU nurses. As listed in Table 2, the univariate regression models revealed significant associations between socio‐demographic and working characteristics and hearing thresholds for: (1) department and (2) working hours. Nurses in the ICU had higher odds of elevated hearing thresholds (univariate OR = 2.45; 95% CI 1.12–5.38) compared to non‐ICU nurses. Also, nurses working 50–59 h per week had higher odds of elevated hearing thresholds (univariate OR = 3.85; 95% CI 1.35–12.88) than those working 40–49 h per week. Those working ≥ 60 h per week had even higher odds (univariate OR = 5.20; 95% CI 1.45–18.65). In the multivariate logistic model, both department and working hours retained significant associations with hearing thresholds. Nurses in the ICU had significantly higher odds (adjusted OR = 2.82; 95% CI 1.25–6.35) than those in non‐ICU. Similarly, nurses working 50–59 h per week had higher odds (adjusted OR = 4.15; 95% CI 1.18–14.60), whereas nurses working ≥ 60 h per week had higher odds (adjusted OR = 5.85; 95% CI 1.55–22.10) than those working 40–49 h per week.

**TABLE 2:** Univariate and multivariate Firth's penalised‐likelihood logistic regression analyses for hearing threshold exceeding the cut‐off (> 25 dB HL) and the socio‐demographic and working characteristics (independent variables) for ICU and non‐ICU nurses (*N* = 93).

	Hearing threshold > 25 dB *n* (%)	OR (95% CI)	$\chi^{2}$	*p*
(a) Univariate model				
Department				
Non‐ICU	5/48 (10.4)	Reference		
ICU	9/45 (20.0)	2.45 (1.12–5.38)	4.73	0.03
Age (years old)				
20–30	4/36 (11.1)	Reference		
31–40	7/47 (14.9)	1.40 (0.58–3.27)	0.34	0.56
> 40	3/10 (30.0)	2.11 (0.78–4.25)	0.68	0.41
Years of working				
< 1	No observations	—		—
1–2 years	2/9 (22.2)	Reference		
3–5 years	2/21 (9.5)	0.48 (0.15–1.55)	1.07	0.30
6–10 years	5/54 (9.3)	0.58 (0.19–1.78)	1.51	0.22
> 10	2/7 (28.5)	1.05 (0.25–4.38)	0.92	0.34
Hours of working				
< 40	No observations	—		—
40–49	3/63 (4.7)	Reference		
50–59	6/18 (33.3)	3.85 (1.35–12.88)	4.71	0.03
≥ 60	5/12 (41.6)	5.20 (1.45–18.65)	6.63	0.01
(b) Multivariate model				
Department				
Non‐ICU	5/48 (10.4)	Reference		
ICU	9/45 (20.0)	2.82 (1.25–6.35)	6.64	0.01
Hours of working				
< 40	No observations			
40–49	3/63 (4.7)	Reference		
50–59	6/18 (33.3)	4.15 (1.18–14.60)	3.96	0.03
≥ 60	5/12 (41.6)	5.85 (1.55–22.10)	5.82	0.01

*Note:* *p*‐values are based on Profile Likelihood Ratio Test (LRT).

Abbreviations: CI, confidence interval; dB, decibel; HL, hearing loss; OR, odds ratio.

The mean PTA (0.5–4 kHz) and the PTA at 8 kHz across different working hours in both ICU and non‐ICU settings were compared in Figure S2. To assess the impact of hearing loss on speech comprehension, weighted calculation method of the average pure tone hearing threshold utilised the following formula: (500 Hz + 2 × 1000 Hz + 2 × 2000 Hz + 4000 Hz)/6. PTA measurements indicate that the hearing thresholds at 8 kHz were worse in those participants who worked longer hours. In particular, nurses working over 60 h per week exhibited significantly higher thresholds (U = 220.5, *p* < 0.001) than those working 50–60 h per week, regardless of whether they worked in the ICU or non‐ICU setting. The hearing thresholds across different age groups in both ICU and non‐ICU nurses were also analysed (see Figure S3). PTA measurements indicate a slight increase in hearing thresholds with age, though the relationship between the PTA and age was statistically significant at 8 kHz (*r* = 0.35, *p* = 0.006) and 0.5–4 kHz (*r* = 0.21, *p* = 0.043) among all nurses. As expected, the age group with the lowest (better) hearing threshold was nurses between 20 to 30 years old. Moreover, ICU nurses consistently reported higher hearing thresholds than non‐ICU nurses in all age groups.

**FIGURE 3:**
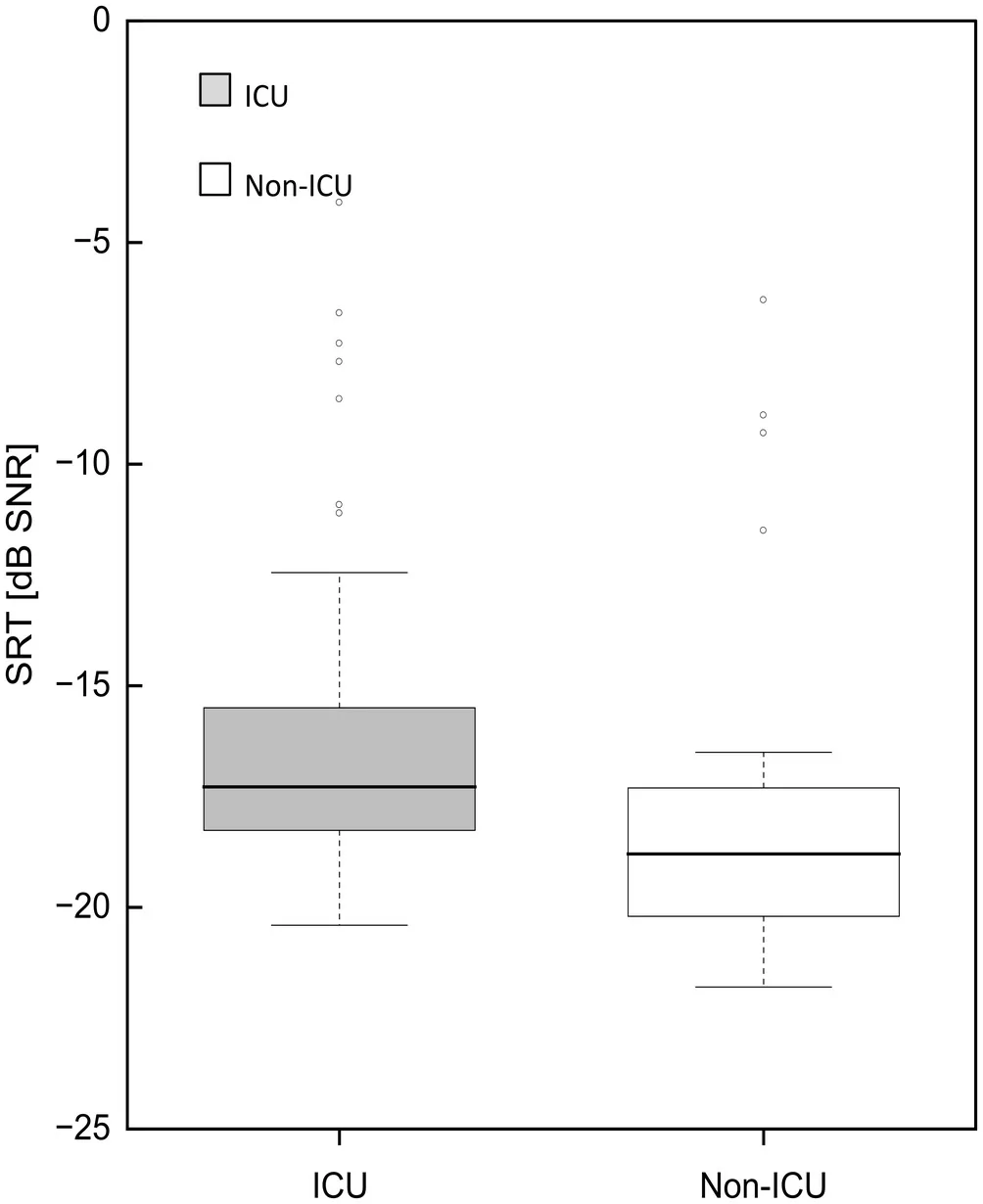
SRT for ICU and non‐ICU nurses. Grey and white boxes indicate ICU and non‐ICU nurses, respectively (SRT: Speech reception thresholds, SNR: Signal‐to‐noise ratio and dB: Decibel).

Figure 3 shows the SRT results from the DIN test for ICU and non‐ICU nurses. The mean SRT was −15.7 ± 4.3 dB SNR for ICU nurses and −18.2 ± 3.2 dB SNR for non‐ICU nurses. On average, the SRT values in both groups were below −15 dB, indicating ‘good’ hearing based on specific cut‐off values. However, 10 individuals in the ICU (22.2%) and six individuals in the non‐ICU (12.5%) were categorised as ‘Needs Help’ in terms of the SRT (> −12.5 dB). It was also observed that ICU nurses exhibited worse SRT than non‐ICU nurses, and the difference between them was significant (*r* = 0.32, *p* = 0.002).

The relationships between SRT and PTA among ICU and non‐ICU nurses under different working durations were analysed (see Figure S4). Consistent with expectations, all data demonstrated significant positive correlations between SRT and PTA (*r* = 0.824, *p* < 0.001), aligning with the theoretical foundation of auditory function assessment. In particular, the types of work (ICU and non‐ICU) had a greater impact on the measured correlation between PTA and SRT than the working hours. ICU nurses exhibited a higher correlation coefficient (*r* = 0.87, *p* < 0.001) compared to non‐ICU nurses (*r* = 0.71, *p* < 0.001). Additionally, nurses working more than 60 h per week (*r* = 0.82, *p* < 0.001) showed stronger correlations than those working 40–50 h per week (*r* = 0.80, *p* < 0.001). The SRT across various working hours for both ICU and non‐ICU nurses were also calculated (see Figure S5). The SRT values increased with extended working hours in ICU nurses and non‐ICU nurses. Specifically, nurses working over 60 h per week exhibited significantly higher SRT (*p* = 0.008) than those 50–60 h per week, especially nurses working in the ICU setting.

## 5 Discussion

The findings revealed that about 20% of the ICU nurses and 10% of the non‐ICU nurses had hearing loss (> 25 dB HL) at high frequencies (4 kHz and 8 kHz). This finding shows a good agreement with the general Chinese population prevalence of 14.2% reported by Bu et al. [19] in Jiangsu, Sichuan, Guizhou and Jilin provinces, suggesting that non‐ICU nurses experience hearing loss at population‐expected rate, while ICU nurses showed higher risk.

The higher prevalence of hearing loss among ICU nurses likely reflects chronic noise exposures in ICU environments. Noise measurements in ICUs consistently recorded 50–70 dBA [20, 21, 22], substantially exceeding the World Health Organisation's recommended maximum of 35 dBA for patient areas. Although extensive research has documented the detrimental effects of this acoustic pollution on patient recovery and sleep [23], the occupational health implications for nurses exposed to these conditions have received limited attention. Several studies have documented high‐noise levels in ICUs [21, 24, 25]. The prevalence of hearing loss among ICU nurses in this study aligns with rates reported in Chinese occupational settings with significant noise exposure—such as metalworking, railroads and packaging industries—which range from 21.3% to 49.0% [26, 27, 28] and is broadly consistent with occupational noise‐induced hearing loss rates reported internationally. Moreover, the high‐frequency hearing loss observed among ICU nurses was consistent with findings in other healthcare professionals and patients across different countries. Messano, Petti [8] reported a 30% incidence of hearing problems among general dental practitioners in Italy, whereas Fujiwara et al. [29] found a 16.8% prevalence of hearing impairment (defined as a > 10 dB increase in PTA thresholds) among ICU patients in Japan. This high‐frequency pattern is further corroborated by Aliyeva, Sari [4], who reported the most significant threshold elevations at 4, 6 and 8 kHz (mean 46.3 ± 15.3, 53.4 ± 11.2 and 57.6 ± 11.0 dB, respectively) in 160 occupationally noise‐exposed workers in Turkey.

Adding to the evidence that elevated high‐frequency thresholds out of proportion with age‐matched averages appears to represent an audiometric signature of noise exposure across clinical and occupational settings. Our study compared the measured hearing thresholds with the international standard (i.e., ISO 7029). The results showed that both ICU and non‐ICU nurses exhibited poorer hearing thresholds than those predicted by the ISO 7029 equation for their respective age groups. This observation aligns with findings from Norway [30] where the median from 51 975 participants also exceeded ISO 7029 thresholds.

ICU nurses reported significantly worse SRT in comparison to non‐ICU nurses. Several factors may explain this difference. First, consistently lower noise levels in non‐ICU environments, as reported in a US hospital study [31], may contribute to better speech communication than in ICU settings. Second, individual differences in auditory function, particularly noise sensitivity and varying degrees of subclinical or noise‐induced mixed and sensorineural hearing loss, may contribute to the observed disparities in speech intelligibility. The clinical significance of impaired SRT in ICU nurses extends beyond personal hearing health. Reduced speech in noise may affect communication with patients, particularly in high‐acuity situations requiring rapid information exchange. Prior research [32, 33] has demonstrated that both psychological sensitivity to noise and physiological hearing impairments can significantly impair speech understanding in adverse acoustic environments. These factors are assumed to account for the variations in speech intelligibilities observed between the two groups. Prior studies have highlighted how individual factors, including noise sensitivity, can affect speech understanding in background noise. Although individual factors might explain a portion of task performance, further research is needed to explore this aspect more comprehensively. Investigating the impact of individual factors would be valuable in a healthcare or ICU setting.

In this study, hearing thresholds from the PTA test were highly correlated with SRT measured through the DIN test. The correlation coefficient between SRT and PTA was approximately 0.8 for all nurses, showing good agreement with earlier research that reported correlation coefficients ranging from 0.66–0.86 [34] and 0.77 [35]. Hearing thresholds at all frequencies significantly contributed to the variance of SRT, with the strongest correlation observed at 2 kHz in both groups. This suggests that speech information near this frequency plays a crucial role in understanding speech in noisy environments, consistent with previous studies [36, 37] demonstrating that 2 kHz covers critical speech formant frequencies.

Interestingly, ICU nurses showed higher PTA‐SRT correlation coefficients (*r* = 0.87) compared to non‐ICU nurses (*r* = 0.71). This discrepancy may stem from the heightened concentration and working memory developed by ICU nurses in their demanding work environment, suggesting that a subject's attitude and concentration may be key factors modulating the SRT‐PTA relationship, with clinical significance for developing targeted hearing protection strategies. Nevertheless, certain limitations of the DIN test need to be considered. First, SRT measurement is influenced by multiple psychological factors such as language proficiency, memory, motivation and concentration [38]. Second, variable effects of hidden hearing loss and signal distortion—potentially reflecting synaptopathy—have been identified in PTA and DIN comparison studies [39].

Extended working hours were identified as a significant independent predictor of hearing impairment, consistent with a clear dose–response pattern. Specifically, nurses working over 60 h per week exhibited significantly higher thresholds (*p* < 0.001) than those working 50–60 h per week, consistent with findings from Park et al. [6] in female workers in South Korea. In this study, approximately 13% of nurses worked more than 60 h per week, representing a particularly vulnerable subgroup. The mechanisms potentially underlying the association between long working hours and hearing impairment include several factors. First, extended exposure to high‐noise levels increases cumulative acoustic dose. Second, stress may exacerbate hearing impairments; given the predominantly female nursing cohort, the additional burden of professional and domestic work may prevent adequate auditory recovery between work shifts [6]. This dose–response pattern is further supported by Aliyeva, Sari [4], who identified job duration exceeding 5 years as a significant independent predictor of noise‐induced hearing loss (NIHL) (adjusted OR = 1.17 per year, *p* = 0.047) in workers in Turkey exposed to sustained occupational noise levels of approximately 90 dB. Taken together, a number of studies are starting to suggest cumulative temporal exposure (whether hours per week or years of service) as a potential risk factor, but further longitudinal studies assessing mediators and confounders will be required to clarify this potential causal link.

Similarly, the number of working hours was significantly associated with SRT in the present study. Nurses working over 60 h per week exhibited significantly higher SRT (*p* < 0.001) compared to those working 50–60 h per week. This result suggests that extended working hours may contribute to fatigue, considering that approximately 13% of nurses in this study worked more than 60 h per week. Park et al. [40] identified 60 h per week as a critical threshold for reversible exhaustion among workers in South Korea, whereas Tucker et al. [41] demonstrated correlations between extended hospital shifts and cognitive fatigue among junior doctors in the UK. These findings suggest that workload reduction and shift scheduling modifications may serve as pragmatic interventions to reduce hearing impairment risk.

## 6 Limitations

Several limitations should be acknowledged for future studies. First, the cross‐sectional design limits our ability to make a causal inference; longitudinal studies are needed to establish temporal relationships between noise exposure and hearing loss. Second, the participant pool was exclusively comprised of nurses from a single hospital, limiting the generalisability to other healthcare workers and geographic regions. Third, this study did not collect personal information regarding noise sensitivity and cognitive ability or impairments. Given that an individual's subjective sensitivity to noise and cognitive functions, particularly after experiencing fatigue, can significantly impact hearing, future investigations need to consider incorporating these individual factors to provide a more comprehensive understanding of the outcomes. Fourth, although Firth penalised regression was employed to address data separation, the 95% confidence intervals for certain estimates remained wide. This reflects the limited statistical power in this subgroup, indicating substantial uncertainty in the effect estimation. Future studies with larger sample sizes are required to validate this finding.

## 7 Implications for Practice and Further Research

These findings highlight direct implications for occupational health policy in hospital settings. Healthcare institutions should implement routine audiological surveillance programmes for ICU nurses, incorporating both PTA and DIN testing to enable early detection and intervention. Beyond standard audiometric frequencies (250 Hz–8 kHz), surveillance protocols should consider including extended high‐frequency audiometry (12–16 kHz) and otoacoustic emissions testing, which can identify outer hair cell damage prior to clinically significant threshold shifts at conventional frequencies [4]. Such enhanced monitoring would enable earlier identification of subclinical auditory damage in ICU nurses before permanent impairment develops. Scheduling reforms to reduce weekly working hours below 50 h per week may also mitigate cumulative acoustic exposure. At an environmental level, hospitals should consider acoustic engineering interventions—such as sound‐absorbing materials, alarm management systems and quieter equipment procurement—to reduce background noise in ICUs to levels closer to WHO recommendations.

Several directions for further research are required. Longitudinal studies are needed to establish causal relationships between ICU noise exposure and progressive hearing loss over nursing careers. Future work should also explore the role of individual factors such as noise sensitivity, tinnitus, cognitive function and fatigue as moderating variables. Crucially, comparative multi‐country studies, including developed countries, would determine whether these findings are generalisable beyond the Chinese context and identify systemic differences in noise regulation, shift patterns and hearing health policy that may inform international occupational health standards.

## 8 Conclusion

This study aimed to investigate hearing problems among nurses in ICU and non‐ICU settings in a single Chinese hospital. The PTA results revealed that ICU nurses had worse hearing thresholds than non‐ICU nurses, particularly at high frequencies. This study also confirmed that the hearing thresholds in ICU and non‐ICU nurses were worse than those expected for their age in the ISO standard (ISO 7029). Furthermore, this study reported the association between average weekly working hours and the risk of hearing impairment at high frequencies, especially in ICU nurses. The DIN tests exhibited a strong and significant positive correlation between SRT and hearing thresholds for both ICU and non‐ICU nurses. The strongest correlation was found at 2 kHz, suggesting that speech information near this frequency is the most important in understanding speech within noisy environments. Moreover, ICU nurses reported significantly worse SRT compared to non‐ICU nurses. This study suggests that nurses, particularly those working for longer hours, in an ICU setting are more likely to experience worse hearing loss than peers working in non‐ICU settings or for shorter hours. These findings are consistent with a dose–response association, highlighting the potential occupational health implications of increased noise exposure in the ICU. Future longitudinal studies are needed to establish causal relationships and to evaluate the effectiveness of targeted hearing conservation programmes for critical care nurses.

## Funding

The authors have nothing to report.

## Ethics Statement

The study was conducted following approval from the Central Ethics Committee of the University of Liverpool (approval number: 11995) and the relevant hospital review board.

## Consent

All participants provided written informed consent prior to enrolment.

## Conflicts of Interest

The authors declare no conflicts of interest.

## Supporting information


**Table S1:** Average hearing thresholds from the PTA test at each frequency, comparing left and right ears among ICU nurses (*N* = 45) and non‐ICU nurses (*N* = 48). Values in parentheses represent standard deviations.
**Figure S1:** Comparisons of hearing thresholds between the current study and the ISO standard for different age bands (top: 20–30 years, middle: 30–40 years and bottom: 40–50 years) (PTA: pure‐tone audiometry, HL: hearing loss, dB: decibel and Hz: hertz).
**Figure S2:** Hearing threshold at different working hours in ICU and non‐ICU settings: (a) PTA at 8 kHz, (b) PTA 0.5–4 kHz. Grey and white boxes indicate ICU and non‐ICU nurses, respectively (PTA: pure‐tone audiometry, HL: hearing loss, dB: decibel and Hz: hertz).
**Figure S3:** Hearing threshold at different ages for ICU and non‐ICU nurses: (a) PTA at 8 kHz, (b) PTA 0.5–4 kHz. Grey and white boxes indicate ICU and non‐ICU nurses, respectively (PTA: pure‐tone audiometry, HL: hearing loss, dB: decibel and Hz: hertz).
**Figure S4:** Relationship between hearing thresholds and SRT results:(a) ICU and non‐ICU nurses and (b) 40–50 h per week and > 60 h per week (SRT: speech reception thresholds, PTA: pure‐tone audiometry, HL: hearing loss, dB: decibel and Hz: hertz).
**Figure S5:** SRT for different working hours for ICU and Non‐ICU nurses (*p < 0.05 and **p < 0.01). Grey and white boxes indicate ICU and non‐ICU nurses, respectively (SRT: speech reception thresholds, SNR: signal‐to‐noise ratio and dB: decibel).

## Data Availability

The data that support the findings of this study are available on request from the corresponding author. The data are not publicly available due to privacy or ethical restrictions.
