# Supplementary material for: Occupational Hearing Loss in the Intensive Care Unit: A Comparative Study Between ICU and Non‐ICU Nursing Staff
Source: Nurs Crit Care. 2026 Sep 24;31(6):e70697. doi: 10.1111/nicc.70697 (PMC13613008; doi:10.1111/nicc.70697)
Supplement: Supplementary file 1 [file NICC-31-0-s001.docx]

**Supplementary Table S1.** Average hearing thresholds from the PTA test at each frequency, comparing left and right ears among ICU nurses (*N*=45) and non-ICU nurses(*N*=48). Values in parentheses represent standard deviations.

|  | ICU nurses (*N*=45) | |  |  |  | Non-ICU nurses (*N*=48) | |  |  |
| --- | --- | --- | --- | --- | --- | --- | --- | --- | --- |
| Frequency [Hz] | Left (SD) | Right (SD) | *t* | *p* |  | Left (SD) | Right (SD) | *t* | *p* |
| 250 | 13 (7.8) | 14 (6.6) | 0.06 | 0.95 |  | 13 (5.7) | 12 (5.2) | 0.03 | 0.98 |
| 500 | 12 (6.6) | 13 (7.6) | 0.03 | 0.98 |  | 12 (6.8) | 11 (6.1) | 0.05 | 0.96 |
| 1k | 13 (7.9) | 12 (8.1) | 0.16 | 0.87 |  | 12 (6.8) | 11 (7.6) | 0.31 | 0.76 |
| 2k | 14 (8.7) | 13 (8.9) | 0.63 | 0.53 |  | 13 (7.3) | 12 (7.8) | 0.46 | 0.65 |
| 4k | 16 (10.6) | 15 (11.5) | 0.46 | 0.65 |  | 15 (7.4) | 15 (8.1) | 0.32 | 0.75 |
| 8k | 19 (10.7) | 19 (12.2) | 0.59 | 0.56 |  | 15 (8.1) | 15 (8.8) | 0.50 | 0.62 |
| Mean PTA  (500, 1k, 2k, and 4k) | 14 (8.2） | 14 (7.8) | 0.18 | 0.86 |  | 13 (6.2) | 12 (6.1) | 0.32 | 0.75 |
|  |  |  |  |  |  |  |  |  |  |

*PTA: pure-tone audiometry, SD: standard deviation, and Hz: hertz)

Supplementary Figure S1. Comparisons of hearing thresholds between the current study and the ISO standard for different age bands (top: 20-30 years, middle: 30-40 years, and bottom: 40-50 years) (PTA: pure-tone audiometry, HL: hearing loss, dB: decibel, and Hz: hertz).

Supplementary Figure S2. Hearing threshold at different working hours in ICU and non-ICU settings: (a) PTA at 8 kHz, (b) PTA 0.5-4 kHz. Grey and white boxes indicate ICU and non-ICU nurses, respectively (PTA: pure-tone audiometry, HL: hearing loss, dB: decibel, and Hz: hertz).

Supplementary Figure S3. Hearing threshold at different ages for ICU and non-ICU nurses: (a) PTA at 8 kHz, (b) PTA 0.5-4 kHz. Grey and white boxes indicate ICU and non-ICU nurses, respectively (PTA: pure-tone audiometry, HL: hearing loss, dB: decibel, and Hz: hertz).

Supplementary Figure S4. Relationship between hearing thresholds and SRT results:(a) ICU and non-ICU nurses and (b) 40-50 hours per week and >60 hours per week (SRT: speech reception thresholds, PTA: pure-tone audiometry, HL: hearing loss, dB: decibel, and Hz: hertz).

Supplementary Figure S5. SRT for different working hours for ICU and Non-ICU nurses (*p<0.05 and **p<0.01). Grey and white boxes indicate ICU and non-ICU nurses, respectively (SRT: speech reception thresholds, SNR: signal-to-noise ratio, and dB: decibel).
